# Multiple Regulatory Mechanisms of Post-Translational Modifications and Therapeutic Potential of Mitotic Catastrophe

**DOI:** 10.3390/ijms27083370

**Published:** 2026-04-09

**Authors:** Qing-Yue Zhang, Xia Chen, Shi-Kun Li, Liang-Zi Cao, Shi-Ying Wang, Ying-Jie He, Xiao-Lin Zhang, Jing-Wei Liu, Xiao-Fang Liu

**Affiliations:** 1 Department of Anus and Intestine Surgery, The First Hospital of China Medical University, Shenyang 110001, China; qyzhang@cmu.edu.cn (Q.-Y.Z.); x22274472@163.com (X.C.); liashtoner@gmail.com (S.-K.L.); 2024110443@cmu.edu.cn (L.-Z.C.); 2 The College of Basic Medical Science, Health Sciences Institute, China Medical University, Shenyang 110122, China; wangshiying1028@163.com (S.-Y.W.); yingjiehe2001@163.com (Y.-J.H.); 3 Department of Pharmacology, School of Pharmacy, China Medical University, Shenyang 110122, China; 18843521573@163.com

**Keywords:** mitotic catastrophe, phosphorylation, ubiquitination, acetylation

## Abstract

Mitotic catastrophe refers to a complicated mechanism of cell death characterized by failure to complete the processes of mitosis correctly due to aberrant chromosome segregation and abnormal tubulin polymerization. Post-translational modifications (PTMs) play a crucial role in the functional diversity of the proteome by mediating the covalent attachment of functional groups to proteins, which regulates the proteolytic cleavage of subunits, facilitating the degradation of entire proteins. Recent studies suggest that PTMs of key proteins are closely implicated in the occurrence, regulation and potential therapeutic targets of mitotic catastrophe. Here, we summarize how multiple PTMs, including phosphorylation, ubiquitination, acetylation, methylation and other types of PTMs, regulate mitotic catastrophe. In addition, potential therapeutic approaches targeting mitotic catastrophe were also discussed. It is anticipated that the inducement of mitotic catastrophe can serve as a promising new therapeutic approach for various diseases in the future.

## 1. Introduction

Mitotic catastrophe (MC) refers to a complicated mechanism of cell death that impedes the proliferation and/or survival of cells that fail to complete the process of mitosis correctly owing to aberrant chromosome segregation, abnormal tubulin polymerization and failure of the mitotic checkpoints. The cellular morphology of MC can be characterized by chromosomal bridges, lagging chromosomes, multipolar mitosis, nuclear membrane rupture, plasma membrane blebbing, nuclear fragmentation, cell swelling and unique nuclear cells (multinuclear, macronuclear and micronuclear) [1].

Post-translational modifications (PTMs) play a crucial role in the functional diversity of the proteome by mediating the covalent attachment of functional groups to proteins, which regulates the proteolytic cleavage of subunits and facilitates the degradation of entire proteins [2]. An increasing number of studies reveal the importance of PTMs in orchestrating dynamic alterations in protein properties and functions, including spatial conformation, enzymatic activity, subcellular localization, folding dynamics, stability and protein–protein interactions, thereby participating in various cellular processes, including signal transduction, gene expression regulation, cell cycle control, DNA repair, apoptosis, mitotic catastrophe and cellular stress responses [3]. The classification of PTMs includes phosphorylation, ubiquitination, SUMOylation, methylation, acetylation, nitrosylation, PARylation, palmitoylation and glycosylation, with each modification possessing unique mechanisms and functions, collectively constituting a complex regulatory network that finely modulates cellular responses to various changes in the environment [4].

Previous studies have shown that dysregulation of PTMs can result in the inability of cells to properly execute mitosis, culminating in mitotic catastrophe. For instance, depletion of histone acetyltransferases reduces acetylated histones, disrupting DNA compaction and organization and ultimately leading to MC [5]. PIAS2β depletion reduces SUMOylation of TUBB3 and PSMC5, disrupting mitotic spindle and centrosome assembly and promoting MC [6]. Further investigation into the mechanisms and applications of MC not only helps to elucidate how cells respond to extreme stress conditions but also offers some new strategies and approaches for cancer treatment.

The interrelationship between MC and PTMs serves to uncover the intricate regulatory mechanisms of cells and provides valuable perspectives on the mechanisms underlying drug resistance in tumor cells and potential therapeutic strategies, yet existing research on their crosstalk remains fragmented and lacks systematic integration. In this review, we summarized different aspects of the interplay between MC and PTMs, including how aberrant PTMs disrupt MC to foster tumorigenesis and how targeting PTMs can reinstate or enhance MC in refractory tumors, as well as the pharmacological implications associated with this relationship. By consolidating these scattered findings into a cohesive framework, this review aims to facilitate the comprehension, accessibility and future investigation of mitotic catastrophe.

## 2. Mitotic Catastrophe and Phosphorylation

**Figure 1:**
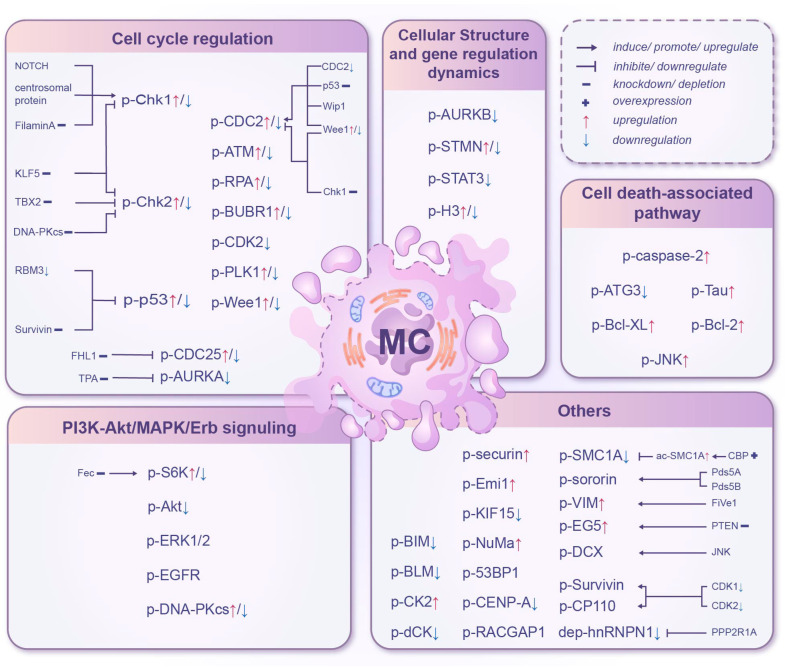
Effects of phosphorylation of multiple substrates on mitotic catastrophe.

Protein phosphorylation is a crucial post-translational modification where a phosphate group is added to specific amino acids of a protein, typically serine, threonine, or tyrosine [7]. This modification can alter the protein’s activity, stability, and interactions with other molecules, thus playing a vital role in regulating various cellular processes [8]. During mitosis, precise protein phosphorylation events are essential for the accurate segregation of chromosomes and the proper progression of cell division (Table 1 and Figure 1). Non-specific effects of the drugs mentioned in this part were also listed in Table 1. These subsections classify phosphorylation’s roles by distinct, non-overlapping cellular processes governing mitosis, from core cell cycle control and death signaling to upstream pathway modulation and structural/epigenetic regulation, each directly linking aberrant phosphorylation to MC.

### 2.1. Cell Cycle Regulation

#### 2.1.1. DNA Damage Response Pathway

DNA damage response orchestrates the processes of DNA repair, cell cycle regulation and apoptosis by recognizing DNA damage signals and activating series of signaling pathways [9]. The signaling pathways include repair pathways, such as Non-Homologous End Joining (NHEJ), Homologous Recombination (HR), Inter-strand Crosslink (ICL) repair and ATM (Ataxia-Telangiectasia Mutated)/ATR (Ataxia-Telangiectasia and Rad3-Related) pathways that regulate cell cycle arrest.

Checkpoint kinase 1 (Chk1) and checkpoint kinase 2 (Chk2), which are phosphorylated and activated in response to DNA damage or replication stress, can phosphorylate downstream substrates to halt the cell cycle for DNA repair [10]. Hyper- or hypo-phosphorylation of Chk1/Chk2 during the arrest period can hinder the progression of the next step in this pathway, which finally culminates in MC [11,12,13]. Against this backdrop, research into novel molecular inhibitors is burgeoning. For example, LY2606308, GNE-900, UCN-01 and C646 induce MC through inhibiting the phosphorylation of Chk1, which entails abnormal degradation of Cdc25A and upregulation of CDK2 [14,15,16,17], whereas XL-844 causes MC by downregulating IR-induced phosphorylation of Chk2 [18]. Likewise, the combination of SNDX-275 and melphalan triggers the upregulation of p-Chk1 and p-Chk2, ultimately leading to MC [19]. In addition, the depletion of specific proteins or the aberrant activation of upstream molecules, such as ATR, can also induce abnormalities in the phosphorylation of Chk1 and Chk2. In Nf1-deficient cells, the suppression of PKC and the phosphorylation of Chk1, both induced by HMG, trigger a persistent mitotic arrest and subsequent MC [20]. Similarly, p-Chk2 can be downregulated by the depletion of TBX2, the DNA-PKcs and centrosomal proteins, all of which are responsible for MC [21,22,23]. The silence of KLF5 and Filamin-A can synchronously inhibit the phosphorylation of Chk1 and Chk2, forcing cells into mitosis with DSBs, which leads to MC [24,25]. Within the ATR-Chk1 signaling pathway, ATR activation induced by NOTCH1 upregulates Chk1 phosphorylation, thereby repressing MC [26]. Conversely, ATR inactivation, induced by ATR inhibitors, downregulates Chk1 phosphorylation, thereby promoting MC [27].

Another guardian of the genome, p53, is a crucial antitumor protein that functions as a transcription factor. P53 maintains genomic stability and cellular health by regulating cell cycle arrest, promoting DNA repair, and inducing apoptosis [28]. The primary site of p53 phosphorylation is Ser-15, which mainly enhance the stability and transcriptional activity of p53 by blocking its interaction with MDM2, consequently inhibiting the degradation of p53. Multiple studies have highlighted the factors contributing to the upregulation of p53 phosphorylation at Ser-15, such as the downregulation of RBM3 [29] and the application of ICF15002 [30]. The upregulation of p53 phosphorylation, which can result in MC due to excessive cell cycle arrest, can also be induced by survivin deficiency and the application of subamolide A [31,32]. In addition, mitotic catastrophe can also be driven by insufficient phosphorylation of p53, which leads to uncontrolled cell cycle progression, as seen in GCTT72 cells where CXCL12 stimulation results in decreased p53 phosphorylation [33]. ATM and ATR are pivotal serine/threonine protein kinases in the DNA damage response pathway, with ATM primarily responding to DSBs while ATR for single-strand DNA damage and replication stress. Oxaliplatin and Rabdocoestin B exhibit antitumor activity by inducing MC through the inhibition of ATM phosphorylation [34,35]. DCZ3301, a novel M phase blocker, enhances the sensitivity of resistant multiple myeloma cells to bortezomib by facilitating the phosphorylation of ATM/ATR, ultimately inducing mitotic catastrophe [36].

Wee1, a pivotal cell cycle regulatory protein kinase, primarily governs cell cycle progression through the catalysis of inhibitory phosphorylation on cyclin-dependent kinases (CDKs) [37]. MS-275, an HDAC inhibitor, suppresses Wee1 phosphorylation by inhibiting HDAC activity, thereby triggering MC [38]. Similarly, resveratrol attenuates the level of Wee1-S642 phosphorylation, which in turn alleviates Temozolomide-induced G2 arrest and leads to mitotic catastrophe [39]. Conversely, withaferin A induces MC by enhancing Wee1 phosphorylation [40].

Replication Protein A (RPA), a trimeric single-stranded DNA-binding protein complex, maintains genomic stability by promoting DNA replication, participating in DNA repair pathways (Nucleotide Excision Repair, Base Excision Repair, HR), and regulating cell cycle and DNA damage responses through interaction and modifications [41]. Upon treatment with BGB324, an AXL inhibitor, Non-Small Cell Lung Cancer (NSCLC) and Large Cell Neuroendocrine Carcinoma (LCNEC) exhibit hyperphosphorylation of RPA at Ser-4 and Ser-8, ultimately leading to MC [42]. In addition, dysregulation of ATR kinase activity induces aberrant phosphorylation of RPA, culminating in mitotic catastrophe [43,44].

#### 2.1.2. G2-M Checkpoint Pathway

Cell Division Cycle Protein 2 (CDC2), also known as Cyclin-Dependent Kinase 1 (CDK1), plays a pivotal role in cell cycle regulation and the mitotic process. The binding of CDC2 to cyclin B is essential for cells to enter mitosis and primarily governs the transition from the G2 phase to the M phase of the cell cycle [45]. The inhibitory sites of CDC2, namely Tyr15 and Thr14, are the main functional phosphorylation sites, the aberration of which can disrupt the proper progression of mitosis [46]. The upregulation of CDC2 phosphorylation at Tyr15 can cause G2/M phase arrest by suppressing the intrinsic activity of CDC2 [47], while the excessive dephosphorylation of CDC2-Tyr15 results in the premature activation of the CDC2-cyclin B complex, which in turn prompts cells to enter mitosis prior to the completion of DNA replication. Both of these alterations can be induced by a variety of factors, which can ultimately trigger the occurrence of mitotic catastrophe. For example, the depletion of Chk1 or the treatment with a Chk1 inhibitor (AZD7762) can downregulate the phosphorylation of CDC2-tyr15 [48,49,50]. Similarly, the inhibition of Wee1 by Wee1 inhibitors (AZD1775/MK-1775 and PD0166285) [49,50,51,52,53,54] or metformin [44], as well as the combined treatment with AURKA or DDK inhibitors and Wee1 inhibitor [55,56] can lead to a reduction in CDC2-tyr15 phosphorylation. The elevation of p-CDC2-Tyr15 can be induced by the upregulation of Wee1 upon treatment with a PLK inhibitor (RO3280) or a PI3K inhibitor, a phenomenon also observed in p53-deficient cells and after WiP1 treatment [57,58,59,60].

Cell Division Cycle 25 (CDC25) facilitates the activation of cyclin-dependent kinases (CDKs) through dephosphorylation, a mechanism crucial for driving the transition between cell cycle phases [61]. Also, the decline in CDC25 phosphorylation can be attributed to several factors, including Chk inhibitors, 8-cl-Ado, UCN-01, and the knockdown of FHL1, all of which lead to the disruption of cell cycle regulation and MC [62,63,64,65]. Moreover, the upregulation of CDC25 phosphorylation induced by DCZ3301 can ultimately lead to MC [36].

Aurora Kinase A (AURKA) is indispensable for orchestrating centrosome maturation, spindle assembly, and the initiation of mitosis [66]. The inhibition of AURKA not only hampers the phosphorylation of KIF15 by AURKA itself but also, when combined with the inhibition of KIF11, exerts a pronounced impact on the spindle assembly, thereby culminating in G2/M phase arrest and MC [67]. Additionally, cells treated with diphenyleneiodonium or depleted in translocated promoter region (TPR) manifest a reduction in AURKA phosphorylation, a change that subsequently leads to MC [68].

Other kinases similarly participate in cell cycle regulation, such as cyclin-dependent kinase 2 (CDK2) and polo-like kinase 1 (PLK1). Dinaciclib, a multi-CDK inhibitor, alters the phosphorylation of CP110 and survivin by inhibiting CDK1 and CDK2 [69]. In addition, the deficiency of FBH1 can facilitate the extent of CDK2 phosphorylation decline induced by Wee 1 inhibitors [70]. Different treatments affect the phosphorylation of PLK1 and consequently suppress its activity to distinct degrees, such as the knockdown of Aurora A/Polo-like-kinase 1 [PLK1]-associated lncRNA (APAL) [71] and the treatment with BI2536 [72] or oxcarbazepine [73]. All these aberrances disrupt the proper progression of the cell cycle, finally culminating in mitotic catastrophe [74].

#### 2.1.3. APC/C-Related Pathway

The Anaphase-Promoting Complex/Cyclosome (APC/C), a multi-subunit E3 ubiquitin ligase complex, plays a central role in cell cycle regulation, involving Cdc27 as a key subunit in the assembly and functional regulation of APC/C [75]. The phosphorylation status of Cdc27 is also associated with MC, as abnormal phosphorylation of Cdc27 can dysregulate APC/C activity, which in turn impairs the spindle assembly checkpoint (SAC) and leads to improper chromosome separation [76]. Emi1, one of the primary inhibitors of APC/C, suppresses its activity by binding to APC/C, thereby blocking its interaction with substrates. Aberrant phosphorylation of Emi1, which compromises its recognition by βTrCP and subsequent degradation, can also result in MC [77]. Bub1-related kinase (BUBR1), a key component of the SAC, interacts with APC/C, a process that is crucial for cell cycle regulation. Similar to trichostatin A, the combination of cisplatin, sodium arsenite, and hyperthermia also induces MC by inhibiting the phosphorylation of BUBR1, which impedes the progression from the G1 phase to the S phase [78,79]. Additionally, the upregulation of BUBR1 phosphorylation triggered by 2-stearoxyphenethyl phosphocholine can further contribute to the induction of MC [80].

### 2.2. Cell Death-Associated Pathway

Mitotic catastrophe is intricately linked to various factors implicated in alternative cell death and senescence pathways, for instance, apoptosis-related factors such as caspase-2, JNK1, and Bcl-2. The over-phosphorylation of caspase-2 induced by AURKB paradoxically exerts an inhibitory effect on its own activity, thereby preventing caspase-2 from cleaving its substrates MDM2 and BID, and ultimately leading to MC [81]. Cells treated with 2-methoxyestradiol induce MC by upregulating the phosphorylation of JNK1, Bcl-2, and Bcl-XL [82]. In addition, it has been reported that the PTK2 inhibitor (PF-573228) can downregulate the phosphorylation of autophagy-related 3 (ATG3), which reduces the degradation of ATG3 and enhances its binding to BAG3, a process that inhibits the function of BAG3 and results in MC [83]. During the progression of cellular senescence, Tau protein is prone to undergo aberrant changes, exemplified by the excessive phosphorylation of Tau protein triggered by okadaic acid, which ultimately can lead to the onset of MC [84,85].

### 2.3. PI3K-Akt/MAPK/Erb Signaling

#### 2.3.1. PI3K-Akt Signaling Pathway

The PI3K signaling pathway, a crucial intracellular signal transduction cascade, maintains cellular homeostasis by modulating a variety of critical processes, including cell growth, survival, and metabolism [86]. As integral members of the PI3K-related kinase family, both DNA-PK and PI3K share a similar kinase domain structure. Specifically, DNA-PKcs, the catalytic subunit of the DNA-PK complex, serves as a central mediator in the repair of DNA double-strand breaks, the regulation of cell cycle checkpoints, and the maintenance of cellular viability. In SKOV3 cells treated with withanolide D and irradiated, the phosphorylation of DNA-PKcs is attenuated, thereby impairing the fidelity of DNA repair processes and ultimately culminating in MC [87]. Similarly, Quercetin inhibits the phosphorylation of DNA-PKcs, consequently disrupting the NHEJ pathway and leading to MC [88]. Conversely, VND3207 can mitigate the occurrence of MC by enhancing the autophosphorylation of DNA-PKcs at Ser-2056 [89].

Akt, alias Protein Kinase B (PKB), and S6K are both pivotal downstream effectors of the PI3K signaling cascade, orchestrating various cellular processes through the intricate regulation of numerous downstream substrates [90]. The mechanism underlying quercetin-induced MC is analogous to that of MG-2477, both involving the inhibition of Akt phosphorylation [88,91]. The deficiency in FES results in an elevation in radiation-induced S6K phosphorylation, whereas AD80 exerts a repressive effect on the phosphorylation of S6K [92,93]. Notably, both of these modulations of S6K-related pathways ultimately converge to induce MC.

#### 2.3.2. MAPK/Erk Signaling Pathway

The Mitogen-Activated Protein Kinase (MAPK) pathway, a highly conserved signaling cascade, is capable of modulating a variety of cellular processes, including proliferation, differentiation, and apoptosis, through phosphorylation events. Extracellular signal-regulated kinase (ERK) is an important branch within the MAPK family. These cascades are specifically triggered by the activation of Epidermal Growth Factor Receptor (EGFR), which acts as a critical upstream regulator to initiate this complex and tightly regulated signaling network [94]. The activation of the ERK1/2 pathway via phosphorylation induced by 2,3,5-tris-(glutathione-S-yl)-hydroquinone (TGHQ) subsequently gives rise to the aberrant phosphorylation of histone H3 and the improper condensation of chromatin, both of which ultimately contribute to MC [95]. AZD6244 or dual inhibition of AURKA and Chk1 can suppress the phosphorylation of ERK, leading to dysfunction of the G2 checkpoint and ultimately resulting in MC [96,97]. Afatinib and selumetinib both can weaken the capacity for DNA repair and predispose cells to MC by inhibiting the phosphorylation of EGFR [98,99].

### 2.4. Cellular Structure and Gene Regulation Dynamics

#### 2.4.1. Microtubule Dynamics

Aurora B kinase (AURKB) activates itself through autophosphorylation, which not only facilitates its binding to substrates but also exerts its influence by phosphorylating these substrates in the regulation of microtubule dynamics [94]. This modulation is crucial for proper assembly and functionality of the mitotic spindle, ultimately ensuring the accurate segregation of chromosomes and the successful completion of cytokinesis. The combined treatment of mulberry fruit water extract (MWE) and IR [100], analogous to the treatment of AdoMet [101], induces cytotoxic effects by inhibiting the phosphorylation of AURKB, thereby triggering G2/M phase arrest and MC. Stathmin (STMN/OP18) is a key regulator of microtubule dynamics, binding to tubulin dimers to inhibit microtubule polymerization and promote depolymerization [102]. Consequently, aberrant phosphorylation of STMN can induce MC by disrupting microtubule stability, for instance, the inhibition of STMN1 phosphorylation caused by CITs [103], while Signal Transducer and Activator of Transcription 3 (STAT3) can influence microtubule dynamics by modulating the expression of various genes associated with microtubule dynamics. The combined treatment of 5-FU and selenetinib downregulates the phosphorylation of STAT3, which in turn leads to a decrease in survivin expression and ultimately results in MC [104].

#### 2.4.2. Epigenetics of Histone H3

Histone H3 phosphorylation is pivotal in mitotic regulation, modulating chromatin condensation, kinetochore stability, and chromosome segregation to ensure proper mitotic completion [105]. Haploid Germ Cell-Specific Nuclear Protein kinase (Haspin) facilitates the proper progression of mitosis by phosphorylating threonine 3 of histone H3 (H3T3), which recruits the Chromosome Passenger Complex (CPC). Given this, the compound CHR-6494, a Haspin inhibitor, downregulates the phosphorylation of H3-Thr3, ultimately leading to MC, an effect also observed with combined Haspin and mTOR inhibition [106,107]. Similarly, the concurrent treatment of DL922 and AZD1152 induces MC by diminishing the phosphorylation of histone H3 [108]. In addition, the phosphorylation of H3 at serine 10 can be downregulated by ophiopogonin B, consequently leading to MC [109], whereas chelidonine exerts an opposing effect by upregulating the phosphorylation of H3-Ser10, ultimately also resulting in MC [110].

### 2.5. Others

The phosphorylation status of other proteins also closely relates to the MC through certain mechanisms. To elaborate, the loss of anillin leads to the suppression of RACGAP1 phosphorylation, which in turn impairs the activation of RhoA and ultimately leads to MC [111]. Similarly, the downregulation of dCK phosphorylation prevents the activation of the G2/M checkpoint, leading to its abrogation and culminating in MC [112]. OAT-449 increases the phosphorylation of NuMa at serine 395, thereby disrupting normal mitotic progression and ultimately inducing MC [113]. Overexpression of CBP enhances the acetylation of SMC1A, which subsequently reduces its phosphorylation and triggers MC [114]. BPR0L075, an anti-microtubule agent, increases the phosphorylation of securin, rendering it unstable and prone to degradation, thereby inciting MC [115]. In osimertinib-resistant EMT cells, the inhibition of AURKB decreases the phosphorylation of BIM at Ser-87, leading to BIM accumulation, abnormal spindle assembly, and increased chromosomal mis-segregation, all of which contribute to the onset of MC [116]. The phosphorylation of sororin induced by pds5A and pds5B results in abnormal sister chromatid separation and ultimately provokes MC [117]. FK228 induces MC by downregulating the phosphorylation of CENP-A, thereby causing abnormal changes in AURKB activity [118]. FiVe1 promotes the phosphorylation of VIM, causing its dysfunction and driving MC [119]. The combined treatment of 212Pb-TCMC-trastumab and paclitaxel downregulates the phosphorylation of CENP-A, finally leading to MC [120]. Abnormal phosphorylation of the protein phosphatase 2A (PP2A) subunit A (PR65) at serine 401 causes chromosomal aberrations, eventually leading to MC [121]. Over-phosphorylation of CK2 disrupts the mitotic process, leading to MC [122]. PTEN deficiency leads to over-phosphorylation of EG5 at threonine 926, disrupting the mitotic process and ultimately causing MC [123]. One mechanism by which roscovitine induces MC is through the dephosphorylation of Bloom syndrome helicase protein (BLM) [124]. PP2A scaffold subunit (PPP2R1A) induces MC by preventing the normal dephosphorylation of hnRNPA1 [125]. Phosphorylation of DCX by JNK results in the persistent activation of kinesin-B, thereby excessively promoting microtubule depolymerization and ultimately leading to MC [126]. 53BP1 can be phosphorylated at serine 25 and serine 1778 by ATM, and this phosphorylation change has an inhibitory effect on MC [127].

## 3. Mitotic Catastrophe and Ubiquitination

Ubiquitination mediates the stability, function, and intracellular fate of proteins by covalently appending ubiquitin molecules to target residues. This process is orchestrated through a sequential enzymatic cascade involving E1 ubiquitin-activating enzymes, E2 ubiquitin-conjugating enzymes, and E3 ubiquitin ligases, of which the E3 ligases specifically recognize the substrate and mediate either mono-ubiquitination or polyubiquitination [128]. The role of ubiquitination in the regulation of the cell cycle, particularly in key events of mitosis such as chromosome segregation and spindle assembly, represents a current focal point of research [129]. This section is categorized by key mitotic functional substrates of ubiquitination, each representing a discrete mitotic process; this classification clarifies how dysregulated ubiquitination of specific mitotic effectors triggers MC.

### 3.1. Cyclin B

As a type of cyclin mainly expressed in G2 and M phases, cyclin B is a key factor in promoting the cell cycle forward and is ubiquitinated by APC/C (an E3 enzyme) for degradation [130]. The HPV-16 E6/E7 proteins can facilitate mitotic entry through enhanced synthesis of cyclin B and promote mitotic exit via ubiquitination-dependent degradation of cyclin B, thereby bypassing checkpoint controls and engaging in aberrant mitosis, ultimately evading MC [131]. In addition, the reduction in cyclin B’s degradation also leads to cell cycle disorder and eventually to MC. Curcumin (diferuloylmethane) inhibits the ubiquitination and degradation of cyclin B, which leads to G2/M phase arrest and ultimately MC [132]. The interaction of MIIP (the migration and invasion inhibitor protein) with Cdc20, a substrate recognition protein in the APC/C pathway, inhibits the APC/C-mediated ubiquitination and degradation of cyclin B and enhances the stability of cyclin B, which in turn triggers MC [133]. Upon paclitaxel treatment, the activated MAD2 (spindle check protein) binds to and thereby impedes the APC/C complex to suppress the ubiquitination and degradation of cyclin B, thus prolonging the activity of the cyclin B/CDC2 kinase complex, which in turn prevents cells from exiting metaphase and culminates in MC [134]. CP5V (a proteolysis targeting chimera) indirectly inhibits the ubiquitination and degradation of cyclin B through the specific ubiquitination and degradation of Cdc20, which interferes with the normal separation of chromosomes and the exit from mitosis, resulting in cell cycle arrest and ultimately inducing MC [135].

### 3.2. Cell Cycle Checkpoints

The cell cycle checkpoint system serves as an intracellular surveillance system, responding to DNA damage or errors in cellular progression by regulating three primary checkpoints: the G1/S checkpoint, the G2/M checkpoint, and the Spindle Assembly Checkpoint (SAC) [136]. The disruption of the D-box in kinetochore protein HEC1 confers resistance to APC/C-Cdh1-mediated ubiquitination and degradation, which subsequently triggers the activation of the mitotic checkpoint and leads to the induction of MC [137]. In addition, the HSP90 inhibitor 17-AAG facilitates the ubiquitination and degradation of BRCA1, thereby impairing the proper activation of the G2/M checkpoint and preventing cells from halting their entry into mitosis, ultimately leading to MC [138].

### 3.3. Aurora Family

The Aurora kinase family consists of three serine/threonine kinases (Aurora-A, Aurora-B, and Aurora-C), which play pivotal roles in cellular division. Within this family, Aurora-A and Aurora-B are central to the orchestration of mitotic events, while Aurora-C exerts distinct functions specifically in meiosis [139]. The inactivation of PKD2 causes the ubiquitination of Aurora A, which inhibits centrosome separation during G2 and significantly increases the cell population harboring unseparated centrosomes, leading to mitotic catastrophe [140]. AZD1152-HQPA inhibits Aurora-B kinase activity and enhances the polyubiquitination and proteasomal degradation of Aurora-B proteins, inducing incorrect chromosome segregation and cytokinesis during mitosis, which exacerbates the occurrence of MC [141].

### 3.4. Others

The impaired ubiquitination and proteasomal degradation of Syk during mitosis impede centrosome function, microtubule organization and spindle formation, ultimately triggering MC [142]. Knockout of DNA-PKcs enhances the ubiquitination of SIK2 and promotes MC by downregulation of C-Nap1, one of the core proteins responsible for centrosome condensation during mitosis [143]. Maintaining the appropriate ubiquitination and degradation of Radmis protein, a microtubule-associated protein (MAP), is crucial for MC, given that elevated Radmis levels disrupt mitotic spindle assembly, whereas its deficiency or downregulation results in the formation of multipolar spindles [144]. Elevated Tripartite Motif 37 (TRIM37) levels promote the ubiquitination and subsequent degradation of pericentriolar material (PCM) proteins, negatively impacting centrosome maturation, microtubule nucleation, and spindle assembly, which culminates in MC [145]. lncRNA Miat augments Sox4 expression by relieving miR-130b-3p-mediated suppression of Sox4, which then diminishes Mdm2-mediated p53 ubiquitination. This cascade of events results in the elevation of p53 downstream factor p21cip1/waf1 expression and the suppression of cyclin B/cdc2 complex activity, ultimately leading to MC [146]. The absence of Swi1 facilitates the ubiquitination and degradation of replication complex components, affecting DNA replication and cell division, finally leading to MC [147]. Inhibition of PTK2 reduces the ubiquitination and degradation of ATG3 (autophagy-related 3), affecting spindle orientation and chromosome segregation, which promotes mitotic catastrophe [83]. Inactivation of Huwe1, a ubiquitin ligase, inhibits H2AX polyubiquitination, causing hyperactivated DNA damage response (DDR), ultimately inducing MC [148]. XAF1 activates E3 ubiquitin ligase XIAP to ubiquitinate and degrade survivin, which triggers MC [149]. When the ubiquitination and degradation of Mcl-1, an anti-apoptotic protein, override a threshold, cells may undergo mitotic catastrophe [150].

## 4. Mitotic Catastrophe and Acetylation

Acetylation, ubiquitous in eukaryotes, involves the addition of an acetyl group to the amino acid residues of proteins [151]. The dynamic equilibrium of acetylation is determined by two types of enzymes: lysine acetyltransferases (KATs), which are responsible for transferring acetyl groups onto amino acid residues, and lysine deacetylases (KDACs), which are tasked with the removal of these acetyl groups. Histone acetylation plays a crucial role in the regulation of gene expression, chromatin structure and cellular function, while non-histone acetylation is involved in key cellular processes such as gene transcription, DNA damage repair and autophagy [152]. Therefore, we will dissect the relationship of acetylation with MC and describe how the imbalance of acetylation levels perturbs mitosis. This section is divided by different enzymes (KATs/KDACs), with each subsection addressing a non-overlapping layer of acetylation control that, when disrupted, impairs mitotic progression and induces MC.

### 4.1. KATs

KATs are primarily categorized into the histone acetyltransferases (p300/CBP, GCN5, MYST) and the non-histone acetyltransferases (p300/CBP, PCAF, SRC-3) [153]. The direct consequence of histone acetyltransferase (HAT) depletion is a decline in acetylated histones, which disturbs the compaction and organization of DNA, leading to a dynamic imbalance and ultimately MC [5]. Curcumin-mediated MC is due to the inhibition of HATs’ activity, which decreases the acetylation level at DSB sites on histones, causing impediments in DNA repair [154]. Similarly, the radio-sensitization effect of C646 is accomplished by inducing MC through the inhibition of p300-HATs, which downregulates the acetylation of histones and drives prematurely repaired DNA damage into the M phase [17]. Furthermore, dysregulation of non-histone acetyltransferases has also been reported to be involved in MC. For example, depletion of nucleolar acetyltransferase NAT10 downregulates the acetylation level of Eg5 at K771, disrupting spindle formation and resulting in the formation of multinucleated giant cells, which leads to MC [155]. And the downregulation of aTATs leads to hypoacetylation of tubulin, thereby affecting microtubule stability and eventually culminating in MC [156]. In addition, the functional impairment of ESCO1/2 markedly diminishes the acetylation of structural maintenance of chromosomes 3 (SMC3), culminating in chromosome arm cohesion defects and ultimately marching towards MC [157].

### 4.2. KDACs

The predominant focus of deacetylases centers on Zinc-dependent histone deacetylases (HDACs), which are categorized into four classes: Class I (HDAC1, HDAC2, HDAC3, HDAC8), Class II (HDAC4, HDAC5, HDAC6, HDAC7, HDAC9, HDAC10), Class III (sirtuins), and Class IV (HDAC11) [158]. The mechanism of HDAC inhibition has emerged as a prominent focus in current research. Inhibition of SIRT1 has been shown to augment the chemosensitivity to cisplatin through the enhancement of MC, which is mediated by increased acetylation at lysine 223 of FOXK2 (Forkhead box K2) [159]. Similarly, the suppression of another sirtuin, SIRT2, elevates acetylation levels of SMC1A at K579, thereby inhibiting the phosphorylation of SMC1A, which triggers aberrant chromosome segregation and the onset of MC [114]. FK228 (depsipeptide) functions as an HDACi that represses the deacetylation of H3K9 (histone H3 lysine 9), which impairs centrosome assembly and subsequently culminates in MC [118]. Likewise, vorinostat inhibits H3 deacetylation, leading to an inability to effectively repair DNA strand breaks and ultimately resulting in MC [160]. In addition, the dynamic equilibrium of acetylated tubulin is crucial in preventing the occurrence of MC. The interaction between SETB1 (SET domain bifurcated 1) and HDAC6 promotes the acetylation of tubulin, subsequently diminishing the stability of microtubules and leading to MC [161]. Furthermore, high concentrations of DTBP (2,4-di-tert-butylphenol) can increase acetylated tubulin through the inhibition of HDAC6, resulting in suppressed microtubule depolymerization and leading to the formation of multinucleated giant cells, which is a morphological hallmark of MC [162]. Another mechanism of Curcumin triggering MC is the inhibition of HDAC4, which increases acetylation of microtubules and arrests cells in the G2/M phase [163].

### 4.3. Others

In addition to modulating enzymatic pathways, a variety of substances and pharmaceuticals can directly or indirectly influence the acetylation of cell cycle-related proteins, thereby mediating the onset of MC. For instance, MC can be triggered by downregulation of SMC3 acetylation levels, instigated by the absence of Pds5 or the inhibition of RIT1, which disrupts the cohesion of sister chromatids and the stability of DNA replication forks [164]. In addition, the upregulation of Nakiterpiosin and filamin A both lead to an increase in acetylated microtubule, resulting in hyperstabilization of microtubule and consequently mediating MC [165,166]. And Miat indirectly promotes the acetylation of p53 to augment the stability of p53 by enhancing the expression of Sox4, which arrests cells in the G2/M phase and ultimately culminates in MC [146]. Safranal, as an aroma in saffron, facilitates the transcription of pro-apoptotic genes by increasing histone H3 acetylation and concurrently hinders the DNA repair process by diminishing histone H4 acetylation. This dual action results in cells’ inability to complete DNA repair, thereby causing MC [167]. Similarly, trichostatin A (TSA) induces MC through the upregulation of H4 acetylation on key cell cycle promoters, leading to cell cycle arrest and cell death [168].

## 5. Mitotic Catastrophe and Methylation

Protein methylation is a reversible enzyme-catalyzed PTM which refers to the process of transferring an active methyl group to the target molecule without altering the DNA sequence composition. Protein methylation occurs in histone and non-histone proteins, which involves in heavy metal modification, regulation of gene expression, processing of ribonucleic acid, and regulation of protein function [169,170]. It has been reported that the aberration within the methylation process can lead to MC. For instance, the combined MLN8237 (Aurora A inhibitor) and chaetocin (HMT inhibitor) treatment synergistically downregulates the levels of H3K9 methylation at centrosomes, which in turn causes aberrant chromosome segregation and ultimately results in MC [171]. The EZH2 inhibitor sensitizes CARM1-high EOCs (epithelial ovarian cancers) to PARPi by inducing chromosomal abnormalities and MC. This process is mediated by the decrease in H3K27me3 on the MAD2L2 gene, the upregulation of MAD2L2 protein, and the subsequent enhancement in error-prone NHEJ activity [172].

## 6. Mitotic Catastrophe and Other Post-Translational Modifications

It has been reported that other PTMs also participate in mitotic catastrophe, for instance, SUMOylation, S-nitrosylation and poly ADP-ribosylation, which play pivotal roles in a variety of cellular processes.

**Figure 2:**
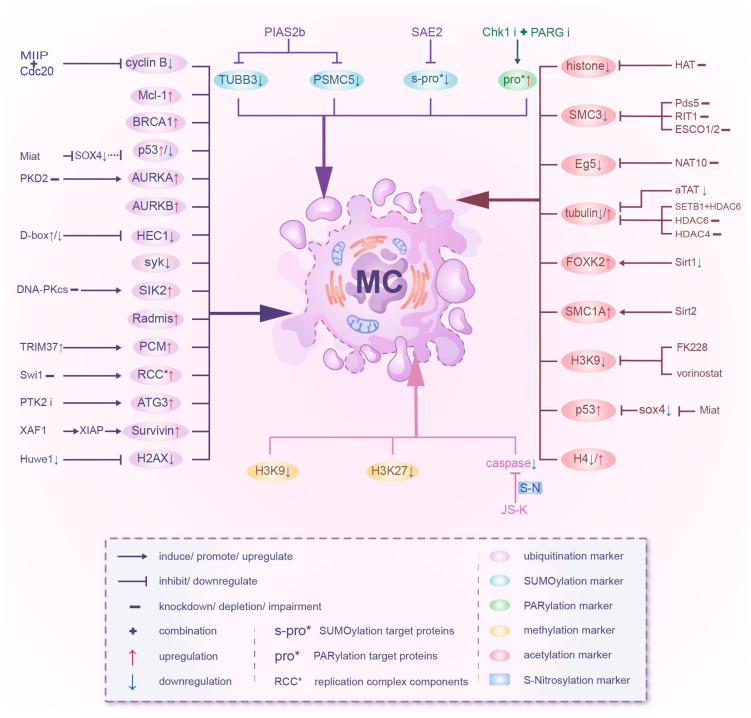
Effects of multiple non-phosphorylation PTMs of different substrates on mitotic catastrophe.

SUMOylation, a process that conjugates the small ubiquitin-like modifier (SUMO) family of proteins to lysine (Lys) residues in target proteins, is a three-step cascade mechanism driven by SUMO-activating enzyme, SUMO-conjugating enzyme and SUMO E3 ligase [173]. The depletion of PIAS2b (a SUMO E3 ligase) promotes MC by downregulating the SUMOylation of TUBB3 and PSMC5, which are required for proper mitotic spindle and centrosome assembly [6]. The inactivation of SAE2 (SUMO-activating enzyme 2) induces a global impairment of SUMOylation in cells with hyperactivated myc, thereby hindering proper mitotic progression and culminating in MC [174]. The NO (nitric oxide) moiety attaches to the thiol group (-SH) of a cysteine residue on a protein through S-nitrosylation, a reversible PTM that is involved in signal transduction, gene expression regulation, apoptosis, and cellular stress response [175]. The NO donor JS-K induces MC by inhibiting caspase activation and modulating cell cycle regulatory proteins via S-nitrosylation [176]. PARylation (poly ADP-ribosylation), using NAD+ as an ADP-ribose donor, is a conserved and reversible PTM that involves the covalent attachment of PAR to target protein by PARP (poly ADP-ribose polymerase) and the removal of PAR by PARG (poly ADP-ribose glycohydrolase) [177,178]. The induction of PARylation by CHK1 inhibitors and the inhibition of dePARPylation by PARG inhibitors synergistically leads to increased replication stress, impaired DNA repair mechanisms, and disrupted cell cycle regulation, ultimately culminating in MC [179] (Table 2 and Figure 2).

## 7. Discussion and Future Perspectives

Mitotic catastrophe is a distinct cell death mode due to unique nuclear changes characterized as multi- or micronucleation. Previous investigations have reported close implications of post-translational modifications of key proteins in mitotic catastrophe. However, a comprehensive understanding of how post-translational modifications modulate mitotic catastrophe remains lacking. Herein, we summarize the molecular mechanisms and potential therapeutic targets of post-translational modifications in regulating mitotic catastrophe.

To date, multiple post-translational modifications have been found to regulate mitotic catastrophe, of which the most frequently reported one is phosphorylation. Protein phosphorylation modulates different signaling pathways, including cell cycle, cell death, PI3K-Akt/MAPK/Erb, cellular structure and gene regulation. Ubiquitination, acetylation, and methylation of core proteins also contribute to the occurrence of mitotic catastrophe, covering multiple aspects of cellular processes. In addition, recent studies suggested that other PTMs also participate in mitotic catastrophe, including SUMOylation, S-nitrosylation and poly ADP-ribosylation. The accurate PTMs of certain signaling molecules are essential for regulating the cell cycle and DNA repair, the aberrant regulation of which might lead to mitotic catastrophe. NuMA is indispensable for establishing and maintaining focused spindle poles during mitosis. Loss of NuMA function impairs stable centrosome–spindle fiber attachment and disrupts sustained focusing of kinetochore fibers at spindle poles. These defects block the formation of stable bipolar spindles to induce G2/M arrest [180,181,182] and mitotic catastrophe [113,183]. It has been reported that polyADP-ribosylation of NuMA [184], NuMA phosphorylation [185], KifC1 (HSET) ubiquitination [186], and Kif18A SUMOylation [187] are critical for normal mitotic processes, including spindle assembly and chromosome segregation; therefore, these PTMs might be implicated in mitotic catastrophe. Cysteine crotonylation (Ccr) of Peroxiredoxin 3 (PRDX3) at the C229 site disrupts dimerization and inhibits redox activity, linking to cellular ROS regulation. Since ROS accumulation triggers spindle disruption, and MC, Ccr may indirectly regulate MC by modulating oxidative stress via PRDX3, offering a new perspective on PTMs and MC [188]. Lactate mediates lysine lactylation (Kla) on 44 histone sites (e.g., H3K23, H4K8) by acetyltransferases like p300. Lactate accumulation induced by hypoxia or bacterial infection can increase Kla levels, which in turn regulate the transcription of genes related to cell cycle progression and DNA damage repair. Thus, Kla may influence hypoxia- or bacterial infection-induced MC through the regulation of G2/M transition-associated genes, a mechanism that requires further verification [189]. β-hydroxybutyrate (Bhb) mediates lysine β-hydroxybutyrylation (Kbhb), a post-translational modification that is markedly upregulated under starvation or diabetic ketoacidosis. Given that Kbhb directly regulates the expression of genes involved in cellular energy metabolism and stress responses, it is highly likely to participate in MC regulation by modulating the transcriptional activity or post-translational modification status of core MC regulatory proteins [190]. The discovery of novel PTMs (Ccr, Kla, Kbhb) reveals a new “metabolite-PTM-gene transcription” axis for stress responses, expanding MC research beyond classical PTMs. In-depth exploration of their effects on MC regulatory pathways will improve the molecular network of MC and provide new targets for developing MC-inducing therapies targeting the PTMs. Additional PTMs and their complex interactions might be revealed by future investigations.

On the basis of the relationships between various PTMs and mitotic catastrophe, multiple therapeutic approaches targeting mitotic catastrophe have been investigated. Studies have indicated that LY2606308, GNE-900, UCN-01 and C646 induce MC through inhibiting Chk1 phosphorylation, while DCZ3301 induces MC by facilitating ATM/ATR phosphorylation. Table 1 systematically clarifies how various pharmacological agents induce MC by regulating substrate phosphorylation. However, most existing research only elucidates the overall effects of these agents on the phosphorylation levels of target proteins, without precisely identifying the specific amino acid residues targeted. This is a limitation for mechanistic research on phosphorylation-mediated MC regulation and its clinical translation. Protein phosphorylation is highly site-specific: phosphorylation of different Ser, Thr or Tyr residues on the same protein leads to distinct conformational changes, molecular interactions and downstream signaling, thus exerting different effects on MC. Thus, clarification of specific target sites is essential for elucidation of molecular mechanisms and potential application of certain drugs. In addition, precise amino acid residue targets are fundamental for rational drug design, as they enable the development of highly specific and high-affinity inhibitors/activators. Therefore, further studies are required to uncover the specific amino acid residues involved in phosphorylation-mediated MC regulation. CP5V inhibits the ubiquitination of cyclin B and induces MC. AZD1152-HQPA enhances the polyubiquitination and proteasomal degradation of Aurora-B protein, inducing incorrect chromosome segregation and the occurrence of MC. As for acetylation, FK228 represses the deacetylation of H3K9 and impairs centrosome assembly, which ultimately results in MC. In addition, DTBP increases acetylated tubulin through the inhibition of HDAC6, resulting in suppressed microtubule depolymerization and MC. Future investigations would reveal more accurate therapeutic targets by the inducement of mitotic catastrophe by regulation of multiple post-translational modifications while minimizing potential side effects. It is anticipated that the induction of mitotic catastrophe can serve as a promising new therapeutic approach for disease therapy in the future.

## Figures and Tables

**Table 1:** Effects of drugs/inhibitors on substrate phosphorylation inducing mitotic catastrophe.

Pharmacological Agents (Drugs/Inhibitors/Combos)	Phosphorylated Substrate	Effect on Substrate	Classic Function
LY2606308	Chk1	−	Chk1 inhibitor
GNE900	Chk1	−	Chk1 inhibitor
UCN-01	Chk1	−	Chk1 inhibitor
C646	Chk1	−	P300 inhibitor
SNDX-275 + melphalan	Chk1	+	HDAC inhibitor
XL-844	Chk	−	Chk inhibitor
ICF15002	p53	+	a melanin-targeted theranostic agent
CXCL12	p53	−	Stromal Cell-Derived Factor 1
PI3K i	CDC2	+	PI3K inhibitor
RO2380	CDC2	+	PLK inhibitor
AZD7762	CDC2	−	Chk inhibitor
AURKA i + DDK i	CDC2	−	AURKA i + DDK i
Wee1 i	CDC2	−	Wee1 inhibitor
metformin	CDC2	−	Non-selective oral hypoglycemic drugs
PD0166285	CDC2	−	Wee1 and PKMYT1 inhibitor
AZD1775/MK-1775	CDC2	−	Wee1 inhibitor
DCZ3301	CDC25	+	M phase blocker
diphenyleneiodonium	AURKA	−	flavoprotein-specific inhibitor
PGE_2_	CDC25	−	Radioprotective
8cl-Ado	CDC25	−	chemotherapeutic agent
Chk i	CDC25	−	Chk1 inhibitor
UCN-01	CDC25	−	Chk1 inhibitor
dinaciclib	Survivin	unknown	Multi-CDK inhibitor
dinaciclib	CP110	unknown	Multi-CDK inhibitor
trichostatin A	BUBR1	−	HDAC inhibitor
Cisplatin + sodium arsenite	BUBR1	−	Cisplatin: Platinum-based chemotherapeutic Sodium arsenite: Antineoplastic agent
2-stearoxyphenethyl phosphocholine	BUBR1	+	Alkylphospholipid antineoplastic
resveratrol	Wee1	−	Natural antitumor agent
MS275	Wee1	−	Class I HDAC inhibitor
withaferin A	Wee1	+	Withanolide antitumor agent
BGB324	RPA	+	Selective AXL inhibitor
DCZ3301	ATM	+	M phase blocker
Oxaliplatin	ATM	−	Platinum-based chemotherapeutic
Rabdocoestin B	ATM	−	Antitumor precursor compound
2-methoxyestradiol	JNK	+	Anti-tubulin anticancer agent
2-methoxyestradiol	Bcl-2	+	Anti-tubulin anticancer agent
2-methoxyestradiol	Bcl-XL	+	Anti-tubulin anticancer agent
PF573228	ATG3	−	PTK2/FAK inhibitor
Okadaic acid	Tau	+	Phosphatase inhibitor

−, an inhibitory effect on the substrate; +, a promoting effect on the substrate.

**Table 2:** Effects of non-phosphorylation PTMs targeting mitotic catastrophe.

PTM Type	Substrate	Modification Site	Effect on Substrate
Ubiquitination	Cyclin B	Lysine residues	−
Ubiquitination	p53	Lysine residues	+
Ubiquitination	BRCA1	Lysine residues	+
Ubiquitination	HEC1	D-box motif	−
Ubiquitination	Cdc20	Lysine residues	−
Ubiquitination	MAD2	Lysine residues	+
Ubiquitination	Aurora A	Lysine residues	−
Ubiquitination	Aurora B	Lysine residues	+
Ubiquitination	Syk	Lysine residues	+
Ubiquitination	SIK2	Lysine residues	+
Ubiquitination	Radmis	Lysine residues	−
Ubiquitination	TRIM37	Lysine residues	+
Ubiquitination	Survivin	Lysine residues	+
Ubiquitination	Mcl-1	Lysine residues	+
S-Nitrosylation	Caspase-2	Cysteine residues	+
PARylation	PARP substrates	ADP-ribose attachment sites	+
Acetylation	Histones	Lysine residues	−
Acetylation	p53	Lysine residues	+
Acetylation	SMC1A	Lysine residues	−
Acetylation	Eg5	Lysine 771	−
Acetylation	Tubulin	Lysine residues	+
Acetylation	SMC3	Lysine residues	−
Acetylation	H3K9	Lysine 9	−
Acetylation	H4	Lysine residues	+
Acetylation	p21	Lysine residues	+
Methylation	Histones	Lysine/arginine residues	−
Methylation	H3K9	Lysine 9	−
Methylation	H3K27	Lysine 27	+
SUMOylation	TUBB3	Lysine residues	−
SUMOylation	PSMC5	Lysine residues	−
SUMOylation	SMC3	Lysine residues	−

−, an inhibitory effect on the substrate; +, a promoting effect on the substrate.

## Data Availability

No new data were created or analyzed in this study. Data sharing is not applicable to this article.
